# Predictive nomogram integrating radiomics and multi‐omics for improved prognosis‐model in cholangiocarcinoma

**DOI:** 10.1002/ctm2.70171

**Published:** 2025-01-12

**Authors:** Yunlu Jia, Mingyu Wan, Yifei Shen, Junli Wang, Xiao Luo, Mengye He, Ruiliang Bai, Wenbo Xiao, Xiaochen Zhang, Jian Ruan

**Affiliations:** ^1^ Department of Medical Oncology the First Affiliated Hospital, Zhejiang University School of Medicine Hangzhou China; ^2^ Department of Laboratory Medicine The First Affiliated Hospital, Zhejiang University School of Medicine Hangzhou China; ^3^ Department of Radiology The First Affiliated Hospital of Zhejiang University School of Medicine Hangzhou China; ^4^ Department of Radiology The Second Affiliated Hospital of Zhejiang University School of Medicine Hangzhou China; ^5^ Interdisciplinary Institute of Neuroscience and Technology School of Medicine Zhejiang University Hangzhou China

Dear Editor,

Intrahepatic cholangiocarcinoma (ICC) is a malignant tumour originating from the epithelial cells of the intrahepatic bile ducts. In recent years, its incidence has shown an upward trend globally. Notably, hepatitis B virus (HBV) infection is one of the significant risk factors for ICC.^1^ Despite significant advancements in medical imaging and molecular biology technologies, predicting the prognosis of HBV‐associated ICC patients remains challenging. One major reason for this challenge is the complex interactions between HBV infection, genetic mutations and tumour behaviour, which increase the uncertainty of prognosis predictions. As a result, traditional single indicators are insufficient for comprehensively assessing patient outcomes. Radiomics is a technology that extracts a large number of quantitative features from medical images, capturing the spatial structure and morphological changes of tumours.^2^ Genomics, on the other hand, focuses on deciphering DNA sequence information, revealing the contributions of genetic variations to disease development. This study aims to develop and validate a predictive model that integrates radiomic features with genomic information. By doing so, it seeks to overcome the limitations of existing biomarkers, better meet the needs for personalised treatment of HBV‐associated ICC patients and provide valuable references for future research and clinical practice.

A total of 389 intrahepatic cholangiocarcinoma (ICC) patients were retrospectively included and divided into a training cohort (210 patients), an internal validation cohort (90 patients) and an external validation cohort (89 patients). Table S1 displayed the clinical and imaging characteristics. The results showed that most clinical characteristics did not differ significantly between the groups, including age (*p* = .188), gender distribution (*p* = .456), the proportion of ferritin ≤323 (*p* = .282), the proportion of high PIVKA‐II (*p* = .988), HBV infection rate (*p* = .158), perineural invasion (*p* = .294) and AJCC 8th edition Classification of Malignant Tumors (TNM) staging (*p* = .455). The only characteristic that showed a statistically significant difference was the presence of vessel cancer embolus (VCE), with the training cohort (19.8%) significantly higher than the internal validation cohort (7.4%) and the external validation cohort (10.4%), *p* = .044. There were no extreme biases between the three cohorts in baseline data. Figure 1 showed the flow diagram of the exclusion criteria of the ICC radiomic datasets. A total of 972 features of magnetic resonance imaging (MRI) images were extracted from the ROIs using the PyRadiomics Python package,^3^ and those with ICC values > 0.8 on both intra‐ and inter‐observer agreement analyses were retained. Table S2 comprehensively summarised the essential patient demographics, such as age, gender and tumour size, along with critical quantitative characteristics extracted from imaging data, including tumour volume and textural features.

**FIGURE 1:**
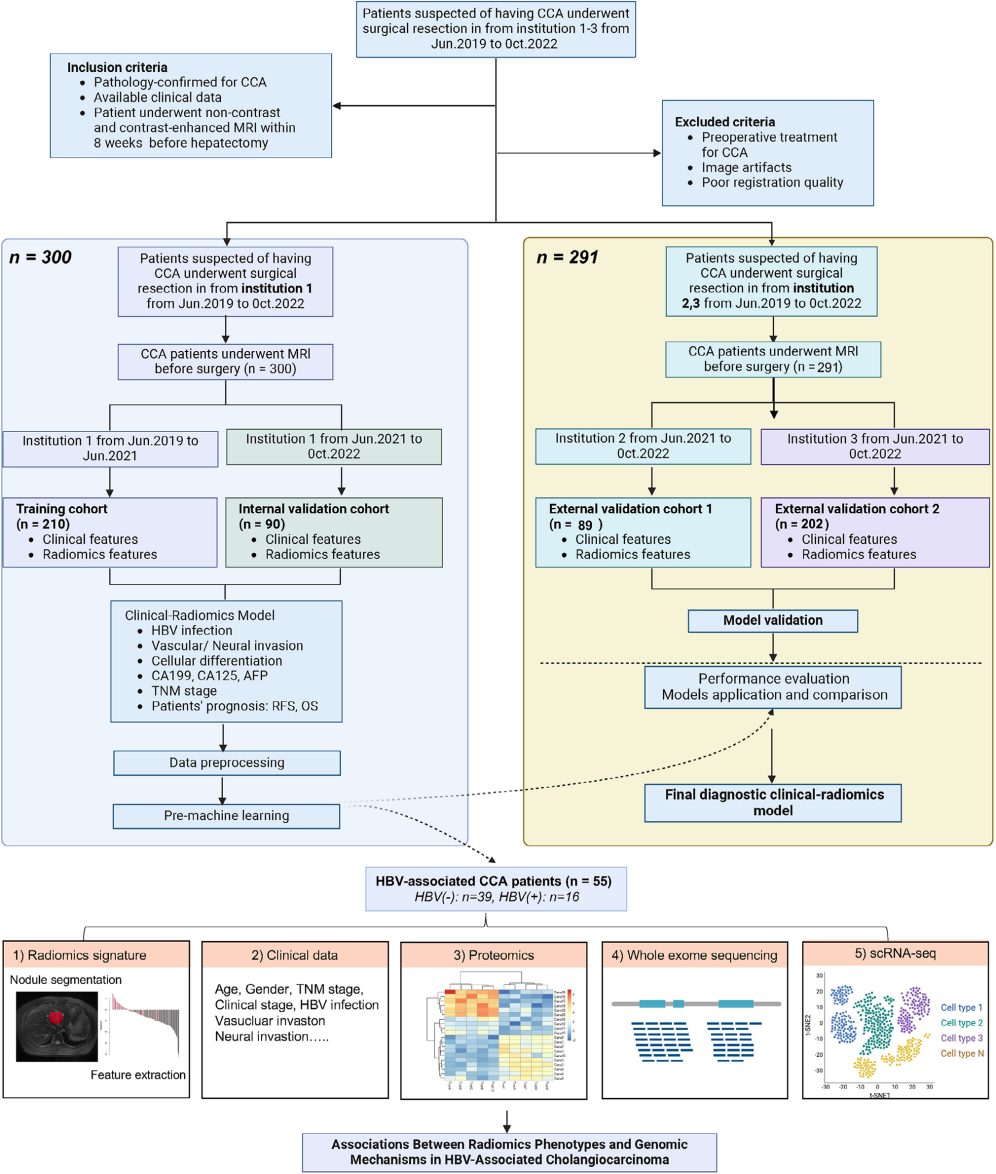
Flow chart summarised the steps performed in this study. A total of 389 patients with histopathologically confirmed ICC were included, coming from three different institutions, and were randomly assigned to a training cohort (210 patients), an internal validation cohort (80 patients), an external validation cohort 1 (89 patients) and an external validation cohort 2 (202 patients). A random forest classifier was applied to analyse radiomic features to predict clinical and pathological factors such as neuroinvasion, CA‐199 levels and HBV infection status. Subsequently, utilising MRI‐derived radiomics, whole‐transcriptome data, scRNA‐seq and machine learning techniques, the study uncovered the relationship between radiomic features and genomic alterations to understand how genetic profiles impact the appearance and behaviour of tumours on medical image. Finally, a nomogram that incorporates radiomics signature and independent risk factors was built to provide a more understandable outcome measure for individualised evaluation, followed by decision curve analysis and survival prediction.

In our study, we evaluated multiple machine learning models to identify the best‐performing algorithm for predicting ICC patient outcomes. Based on our analysis, random forest (RF) emerged as the top performer due to its ability to handle high‐dimensional data and capture complex interactions, which are common in genomics and proteomics datasets. Its robustness against overfitting and provision of feature importance scores were particularly advantageous for our multi‐omics approach. While support vector machines (SVM) showed strong performance, especially in high‐dimensional spaces, the computational cost and interpretability challenges made it less favourable for our specific application. Logistic regression (LR), although simpler and more interpretable, did not capture the complexity of the data as effectively as RF. In the training cohort, radiomic models were capable of predicting the pathological factors, including HBV infection, cell differentiation, CA19‐9 and neuro invasion (Figures 2A and S1A). Overall, the RF model performed the best in the training cohort, but its performance in the external validation cohort may have been affected by changes in data distribution. To address this issue, we have added a new external validation cohort 2 containing 202 patients. This larger sample size helped to better capture the underlying data distribution and reduce the risk of overfitting. Additionally, we have refined our analysis methods to include additional regularisation techniques and hyperparameter tuning, enhancing the robustness and reliability of our model. As a result, the area under the curve (AUC)values of the RF model for the new external validation cohort have shown some improvement, although they were still lower than those in the training cohort. This suggested that while overfitting was a concern, the model's performance can be improved with a larger and more diverse validation set. Detailed information including threshold, sensitivity, specificity, accuracy, precision and evaluation statistics from three model construction were presented in Table S3. Subsequently, we delved into whether radiomic models could predict somatic mutations in the most frequently mutated genes in ICC, specifically *KRAS, BRAF, FGFR2* and *IDH1/2*.^4^ Our results highlighted that among these, *FGFR2* mutations were predicted with the utmost accuracy, achieving an AUC value of 0.86 (Figure S1B). While the predictive performance for other genes, such as *KRAS* and *BRAF*, may not have been as prominent as that for *IDH1/2 and FGF*R2, which both achieved an AUC value of 0.86. Despite its moderate performance in predicting specific gene mutations such as *KRAS* and *FGFR2*, the radiomics‐genomics model retains significant clinical utility. When the model indicates a higher likelihood of these mutations, clinicians can incorporate this information into their treatment planning processes and refine individualised therapy strategies following comprehensive genetic testing. Moreover, radiomics features associated with these gene mutations can serve as auxiliary tools for evaluating ICC patient prognosis.

**FIGURE 2:**
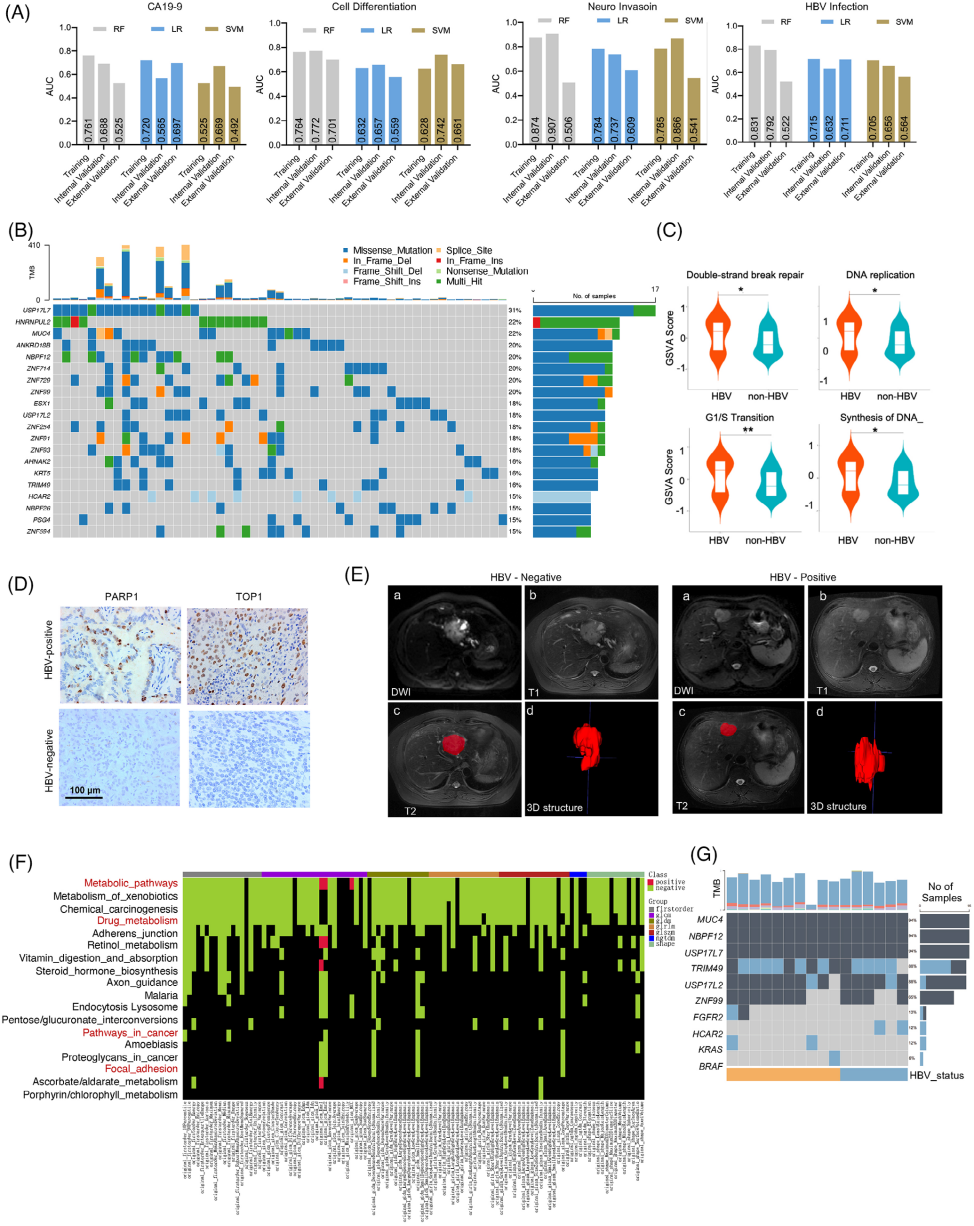
Correlation matrix between radiomic phenotypes and genomic markers in HBV‐associated ICC. (A) Radiomics models, including random forest (RF), logistic regression (LR) and support vector machines (SVM), were capable of predicting pathological factors such as HBV infection, cell differentiation, CA‐199 level and neuro‐invasion in the training, internal validation and external validation ICC cohort. (B) Prevalence of genetic variations among 55 ICC samples, with 96.36% exhibiting variations including missense mutations, in‐frame deletions and insertions, frame‐shift alterations, splice‐site variants, nonsense mutations and multiple hits. (C) Proteomic‐based gene set enrichment analysis revealing significant upregulation of oncogenic pathways, including DNA replication, cell–cell junction organisation, cell cycle and DNA repair, in HBV‐positive ICCs. (D) Immunohistochemistry validation of the expression of DNA repair‐related genes PARP1 and TOP1 exclusively in HBV‐positive tumours. (E) Case examples of patients with tumours in the oropharynx, one HBV‐positive (HBV+) and one HBV‐negative (HBV−), accurately predicted by the radiomic model. (F) Illustration of the connections between radiomic features and cancer‐related KEGG pathways. Radiomic features were associated with multiple aspects of the cancer molecular system, including metabolic pathway, signal transduction, cell growth and death, immune system and cellular interactions and communication. This analysis suggests the possibility of indirectly gaining insight into the tumour's molecular activities through imaging features. (G) Evaluation of the predictive capability of a machine learning‐based radiomics model. MRI data from an external validation set of ICC patients were used to identify cases predicted to harbour gene mutations. WES analysis of these predicted cases revealed high frequencies of specific gene mutations, including MUC4, NBPF12, USP17L7 and TRIM49, demonstrating the potential of the radiomics model in accurately predicting gene mutations non‐invasively.

We conducted a comprehensive genetic and proteomic characterisation of ICC samples based on HBV infection status. The results highlighted the prevalence of genetic variations among 55 ICC samples, with 96.36% exhibiting variations, including missense mutations, in‐frame deletions and insertions (Figure 2B). The most frequently detected driver gene variants associated with HBV status were *USP17L7*, *MUC4*, *USP17L2*, *ZNF99*, *HNRNPL2*, *NBPF12*, *HCAR2* and *TRIM49* (Figure S1C). The Table S4 presented a comparison of variant frequencies between HBV‐negative and HBV‐positive samples. While there were notable differences in variant frequencies for some genes, such as *USP17L7* and *HCAR2*, between the two groups, the majority of genes exhibit statistically insignificant differences in their variant frequencies. In the TCGA database, the mutation rate of the *MUC4* gene was recorded at 21%. MUC4 was a mucin whose aberrant expression was associated with various cancer types, including ICC.^5^ The high mutation rate suggested that MUC4 may play a significant role in the pathogenesis of ICC (Figure S1D). In addition, ICC patients harbouring mutations in these genes exhibited higher tumour mutation burdens and mutation counts,^6^, ^7^ which suggested that mutations in these genes may be closely linked to genetic instability and the process of tumour evolution in ICC (Figure S1E). Proteomic‐based gene set enrichment analysis revealed that oncogenic pathways, including DNA replication, cell–cell junction organisation, cell cycle and DNA repair, were significantly upregulated in HBV‐positive ICC (Figures 2C and S1F). Particularly, the expression of DNA repair‐related genes *PARP1* and *TOP1* was identified exclusively in HBV‐positive tumours, which were validated through immunohistochemistry (Figure 2D). The upregulated DNA repair pathways may render HBV‐positive ICC cells dependent on specific repair mechanisms. For example, PARP inhibitors have been successfully used in BRCA1/2‐deficient tumours, and this strategy can be extended to other cancer types with upregulated DNA repair pathways. The effects of novel damage response (DDR) inhibitors, such as ataxia telangiectasia mutated (ATM) kinase inhibitor, on HBV‐positive ICC could be tested using preclinical models or clinical trials. Differential gene expression analysis identified 672 proteins that were significantly over‐represented in HBV‐positive ICCs (fold change > 2; adjusted *p* < .05; Table S5).

To gain insights into the potential relationship between HBV status and clinical pathological as well as radiomic features, we applied the correlation analysis. As presented in Table S6, some radiomic features, including shape elongation and certain parameters of the grey‐level co‐occurrence matrix, exhibited significant correlations with HBV status, providing data supported for further exploration of the impact of HBV on imaging features. To further interpret the radiomics models pertaining to HBV, we presented two case examples of ICC patients. Our model accurately predicted the HBV status of these patients (Figure 2E). The predictions were based on a set of radiomic features extracted from the segmented MRI images. Specifically, these features included characteristics such as shape elongation and certain parameters of the grey‐level co‐occurrence matrix, which have been shown to differ significantly between HBV‐infected and non‐infected individuals. By leveraging these distinct imaging biomarkers, the model can differentiate between HBV‐positive and HBV‐negative statuses. Next, we applied the RF classifier to predict HBV status on the basis of tumour radiomic features. Our radiomic models demonstrated a significant ability to distinguish HBV‐positive from HBV‐negative status (training cohort AUC = 0.73). However, to provide a more comprehensive assessment of the model's performance, we also evaluated it using an independent internal validation cohort (*n* = 90; AUC = 0.64) and an external validation cohort (*n* = 89; AUC = 0.65). These results indicated that, although there was a minor reduction in performance, the model retained its predictive capability across different patient populations (Figure S1G). We also provided a comprehensive baseline statistical analysis of relevant imaging features in samples stratified by HBV infection status (Table S7). In the training cohort, significant differences were observed in multiple imaging features between HBV‐infected and non‐infected individuals, including Elongation, Maximum 2D Diameter Column, GLCM Correlation and GLCM Idm. However, the *p* values for these features in the internal and external validation cohorts, including a new external validation set of 202 cases, did not show significant improvement, suggesting that further validation was needed to confirm their robustness and generalisability. This predictive outcome was consistent with clinical test results, indicating that radiomics features can effectively reflect the HBV infection status of tumours, offering the potential for non‐invasive diagnosis of HBV‐related tumours.

HBV infection may affect the metabolic pathways of hepatocytes, leading to abnormalities in glycogen synthesis and breakdown, as well as fat metabolism.^8^ These changes may manifest radiologically as hepatic steatosis or inhomogeneity in density.^9^ Here, we found that radiomic features were associated with cancer‐related KEGG pathways covering multiple aspects of the cancer molecular system (Figure 2F). Specifically, transcriptional activity of several molecular signalling pathways, including chemical carcinogenesis, drug metabolism was negatively associated with tumour size features, indicating that they were more active in smaller tumours than larger tumours. In addition, *MUC4* and *USP17L7* mutations were highly correlated with HBV status, and patients with HBV‐infected ICC often exhibited low LRBA, MUC5AC and MUC1 expression (Figure S1H). In order to evaluate the predictive capability of a machine learning‐based radiomics model, we extracted MRI data from a cohort of ICC patients within an external validation set and identified cases predicted to harbour gene mutations, yielding a total of 16 subjects (Figure 2G). Subsequent WES analysis revealed that the frequencies of gene mutations among these 16 patients in the validation set were as follows: *MUC4*: 94%, *NBPF12*: 94%, *USP17L7*: 88% and *TRIM49*: 88%. These results demonstrated the promising potential of this radiomics model in accurately predicting gene mutations without invasive procedures, warranting further investigation on a larger scale to validate its efficacy.

We next conducted scRNA‐seq experiments on samples from three ICC patients, comprising one HBV‐ICC patient (ICC 1) and two non‐HBV‐ICC (ICC 2 and ICC 3) patients. By examining cell‐type‐specific markers, tumour‐associated markers and copy number variations (CNVs) within individual cells, we were able to definitively identify the specific types of different cells (Figure 3A). Compared with HBV‐negative samples, HBV‐positive samples have fewer cell–cell interaction types and stronger interaction strength (Figures 3B and S2A) Detailed changes in the number and strength of intercellular interactions among samples with different HBV statuses were shown in Figure 3C,D. Nine functional groups were significantly enriched in CD4+ and CD8+ T cells (*p* ≤ .05), including somatic cell DNA recombination, regulation of DNA recombination, DNA recombination and DNA deamination (Figure 3C), which was consistent with the results of GO term pathway analysis (Figure 3E). The observed low and significant expression of GSTP1 and TOP1, along with a similar but not significant trend for ATM, suggested alterations in various processes critical for maintaining genomic stability (Figures 3F and S2B). Next, the pseudotime analysis revealed consistently low expression levels of GSTP1, TOP1 and ATM, with only slight increase observed over time in HBV‐positive ICC (Figure 3G). These findings provided valuable insights into the molecular mechanisms and cellular interactions that distinguish HBV‐ICC from non‐HBV‐ICC, highlighting the importance of CD8+ T cells in specific molecular processes and the potential roles of GSTP1, TOP1 and ATM in DNA replication and repair within HBV‐ICC.

**FIGURE 3:**
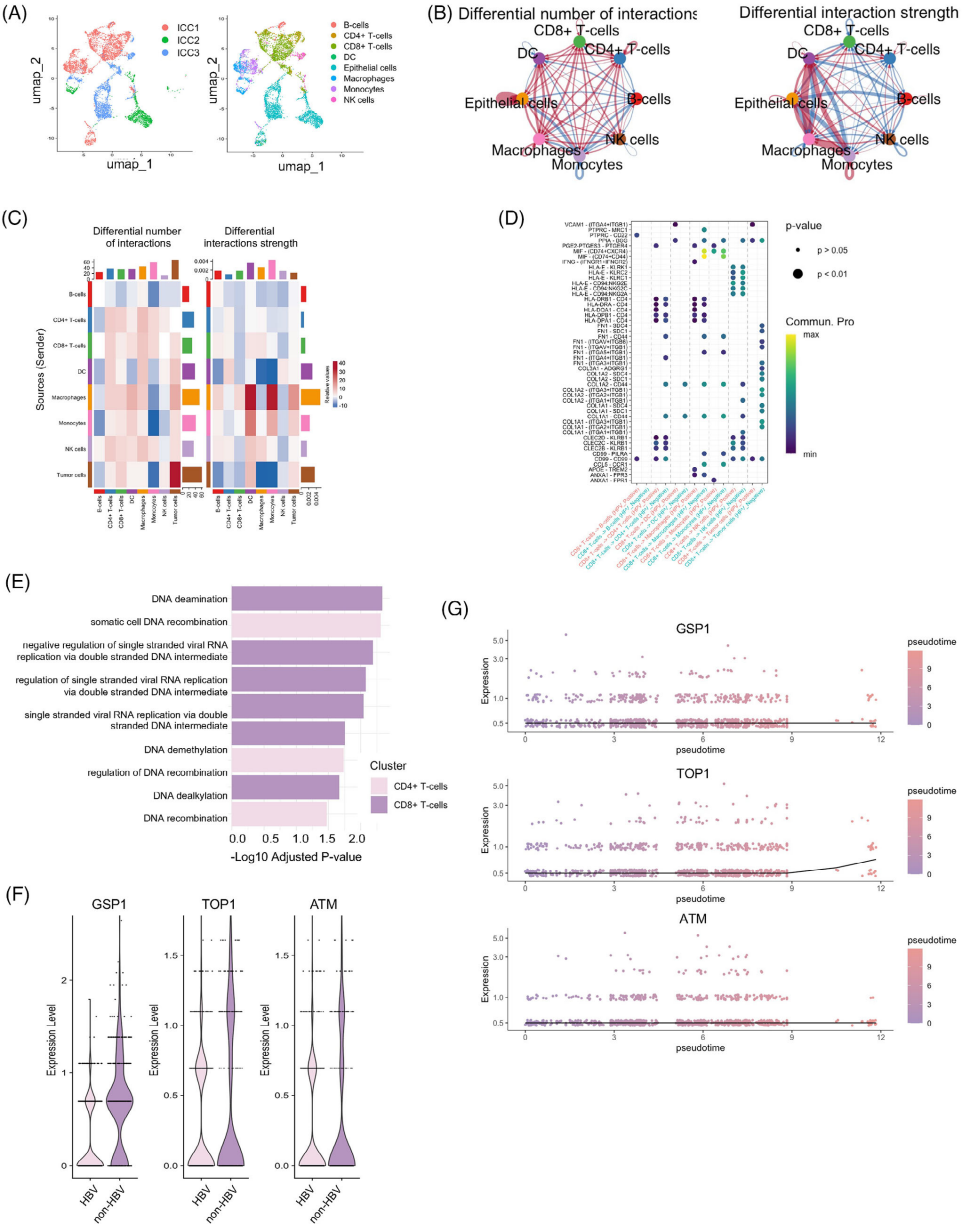
Single‐cell RNA sequencing of HBV‐negative and HBV‐positive ICC. (A) Cell‐type identification based on specific markers, tumour‐associated markers and copy number variations (CNVs) within individual cells. (B) CellChat analysis showing the differential number and strength of intercellular interactions in HBV‐positive samples compared with HBV‐negative samples, based on upregulated ligand‐receptor pairs. (C) Detailed changes in the number of intercellular interactions among samples with different HBV statuses. The plot illustrates the specific alterations in interaction frequencies between various cell types in HBV‐ICC versus non‐HBV‐ICC samples. (D) Detailed changes in the strength of intercellular interactions among samples with different HBV statuses. (E) Functional enrichment analysis of CD4+ and CD8+ T cells, highlighting significantly enriched GO terms related to DNA recombination, repair and regulation. (F) Relative expression levels of GSTP1, TOP1 and ATM in sample ICC1, visualised on UMAP coordinates. (G) Pseudotime analysis showing consistently low expression levels of GSTP1, TOP1 and ATM in HBV‐positive ICC, with slight increases observed over time.

Three types of OS models were developed, including a pathology‐based model, an imaging‐pathology based model and an imaging‐pathology‐genomic based model (Figure S3A,B). The imaging‐pathology based OS model also included several predictors, including Radscore derived from arterial phase imaging, pT stage, pN stage, CA125, CA199 and CEA level, with a model cutoff value of 193.49. Based on the cutoff value of 193.49, this model categorised ICC patients into a high‐risk group (median value of Risk2: 304.1, interquartile range (IQR): 210–447) and a low‐risk group (median value of Risk2: 142.4, IQR: 67–165.89) (*p* < .0001) (Figure 4A). Finally, the calibration curve of the imaging‐pathology‐based OS model demonstrated good agreement between predictions and observations (Figure 4B). In predicting the OS of patients with ICC, the imaging‐pathology based OS model demonstrated the best performance. Specifically, this model had the highest C‐index and the lowest p‐value, showcasing its superior capability in predicting patient prognosis.

**FIGURE 4:**
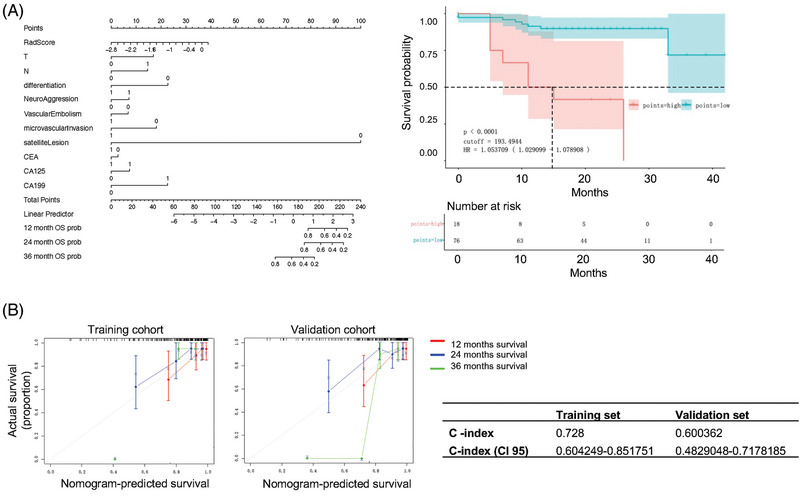
Construction of pathology‐radiomics nomogram for predicting ICC patient prognosis. (A) The imaging‐pathology‐based OS model presented with a nomogram scaled by the proportional regression coefficient of each predictor. Kaplan–Meier survival curves for the imaging‐pathology‐based OS model. Patients were stratified into high‐risk and low‐risk groups based on the model cutoff value. The 1‐year, 2‐year and 3‐year survival rates were significantly different between the two groups. (B) Calibration curve for the imaging‐pathology based OS model. The curve shows good agreement between the predicted and observed probabilities of OS, indicating the model's reliability in predicting patient prognosis.

Utilising radiomics derived from MRI, whole‐transcriptome data and machine learning techniques, this is the first study combining radiomic features from MRI images with full‐genome measurements that depict the multi‐layered tumour molecular systems in ICC. Our radiogenomics nomogram provides a powerful and non‐invasive method for predicting ICC prognosis, thereby supporting more informed clinical decision‐making and facilitating personalised treatment approaches. The future integration of radiomics with deep learning holds significant potential to revolutionise medical diagnostics and personalised medicine. By leveraging multi‐parametric imaging frameworks, such as MPRAD, this integration combines information from multiple imaging sequences to provide more comprehensive diagnostic data. Deep learning algorithms can automatically uncover complex patterns within these images, thereby enhancing predictive accuracy and aiding in the discovery of subtle disease indicators. Future research can extend to larger and more diverse ICC patient populations to provide more robust model validation, and through multi‐centre collaborations, achieve the integration of resources and expertise, ensuring the reproducibility of results.

## LIMITATIONS

Despite the multiple measures we took in the study design and data analysis to ensure the reliability and validity of the results, several limitations remain in this study. First, there is a discrepancy in the incidence of VCE between the training and validation sets, which may affect the model's generalisability. This difference is likely due to the inherent variability in patient recruitment periods and sources. Future studies will aim to reduce these differences by increasing the sample size and using more consistent recruitment criteria. Besides, the moderate AUC of HBV prediction observed in external validation indicates that while our model performs reasonably well within its original context, its generalisation across different populations or settings may be less effective. This limitation can be attributed to data heterogeneity arising from differences in data collection methods and patient demographics between the training and external validation sets, or cohort variability due to variations in clinical characteristics and treatment regimens. To enhance the model's generalisability in future research, we plan to incorporate more diverse training data from multiple centres and regions to capture a broader spectrum of variability, thus enhancing the model's robustness.

## AUTHOR CONTRIBUTIONS

Y. Jia, M. Wan, Y. Shen, and J. Wang analyzed the data in the study and, as co‐first authors, contributed equally to this work. X. Luo and M. He participated in the data collection and provided critical feedback that shaped the final research outcomes. R. Bai and W. Xiao contributed to the data collection and offered essential feedback. X. Zhang and J. Ruan designed and supervised the study and provided critical feedback at all stages of the research. All authors provided critical feedback and helped shape the research, analysis and manuscript.

## CONFLICT OF INTEREST STATEMENT

The authors declare no conflict of interest.

## Supporting information



Supporting Information

Supporting Information

Supporting Information

Supporting Information

Supporting Information

Supporting Information

Supporting Information

Supporting Information

Supporting Information

Supporting Information

Supporting Information
