# Supplementary figures and images for: Predictive nomogram integrating radiomics and multi‐omics for improved prognosis‐model in cholangiocarcinoma
Source: Clin Transl Med. 2025 Jan 12;15(1):e70171. doi: 10.1002/ctm2.70171 (PMC11726632; doi:10.1002/ctm2.70171)

Supplementary Figure 1

A

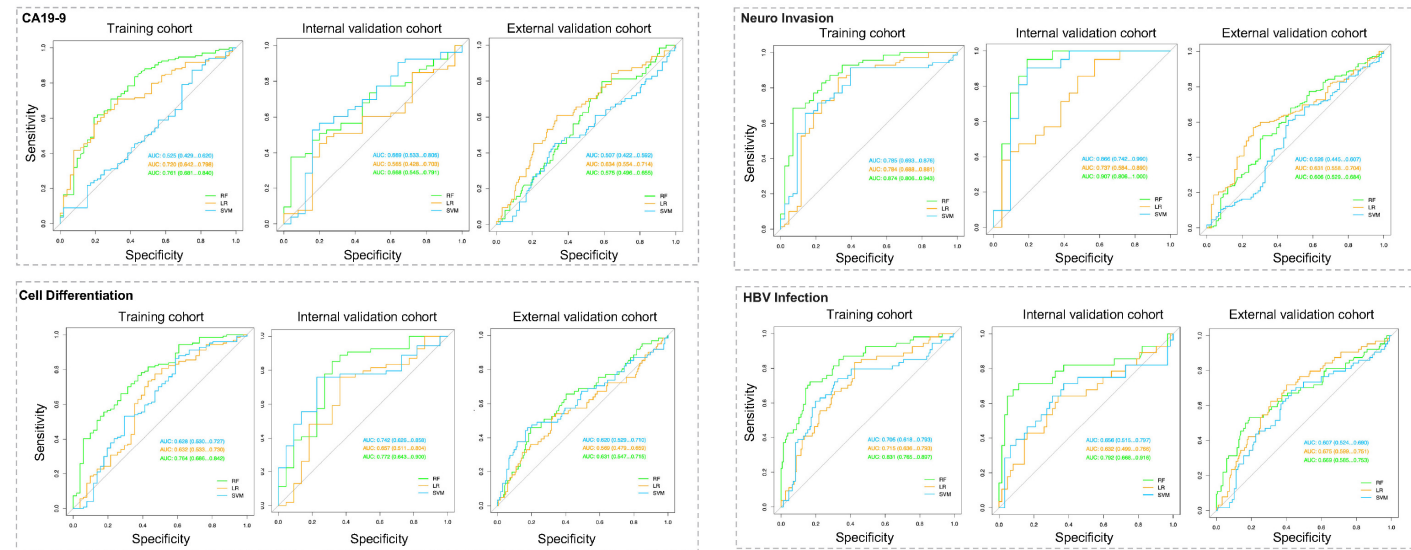

B

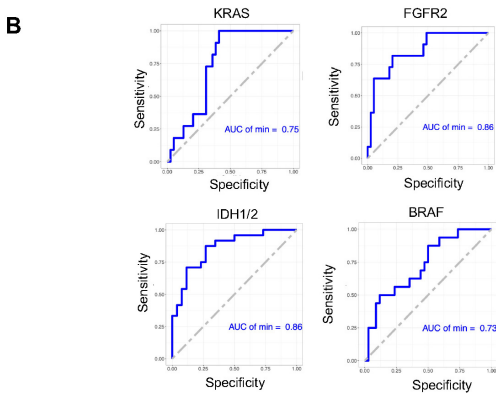

D

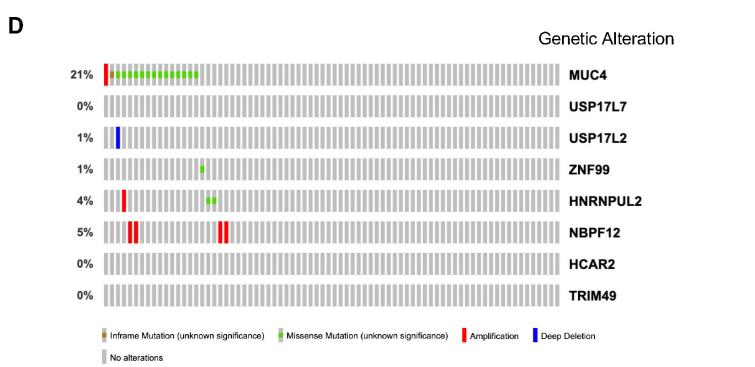

C

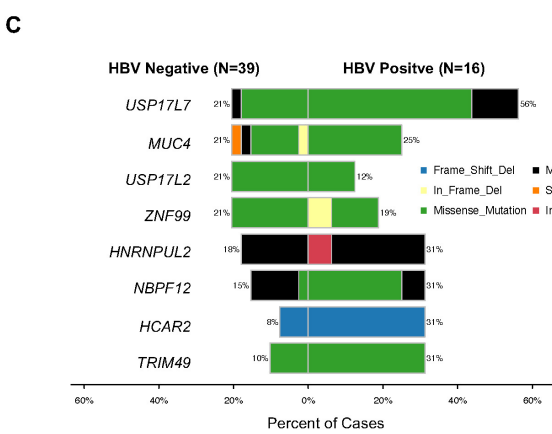

E

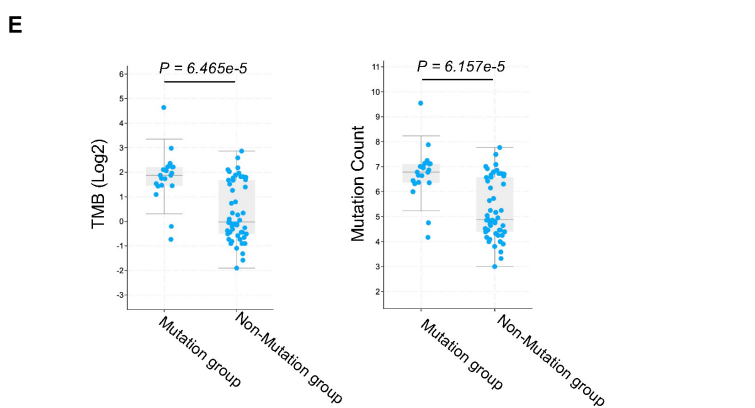

F

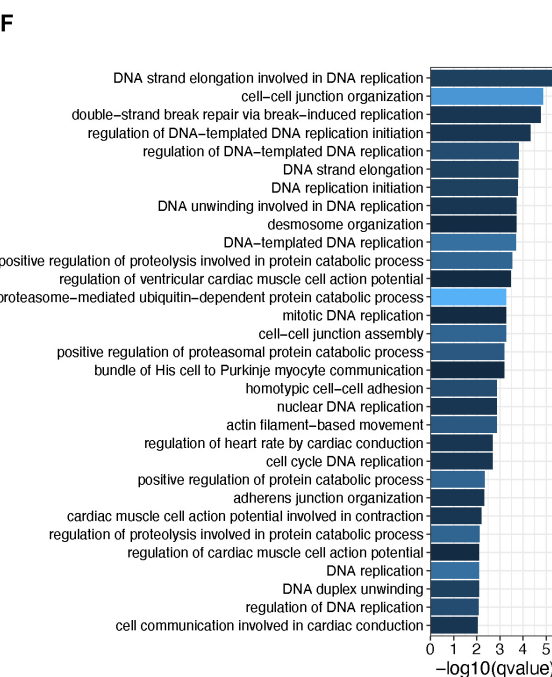

G

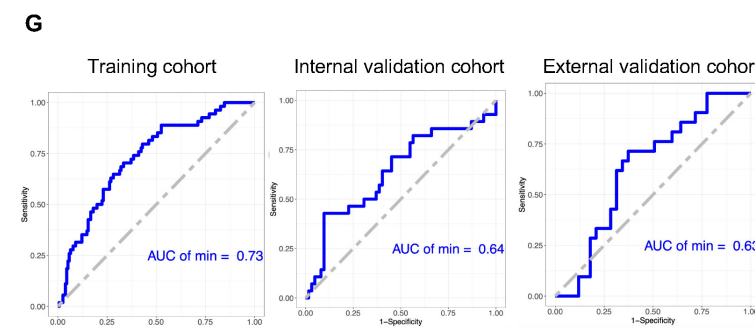

H

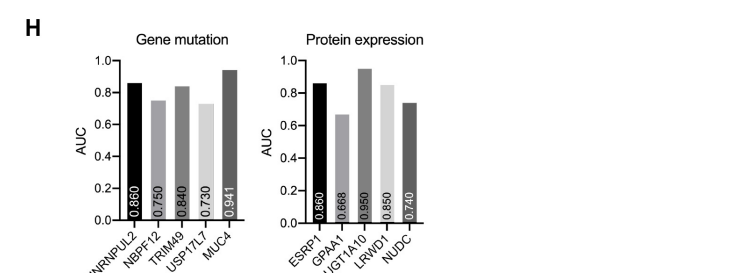

Supplement: Supplementary file 1 — Supporting Information [file CTM2-15-e70171-s002.pdf]

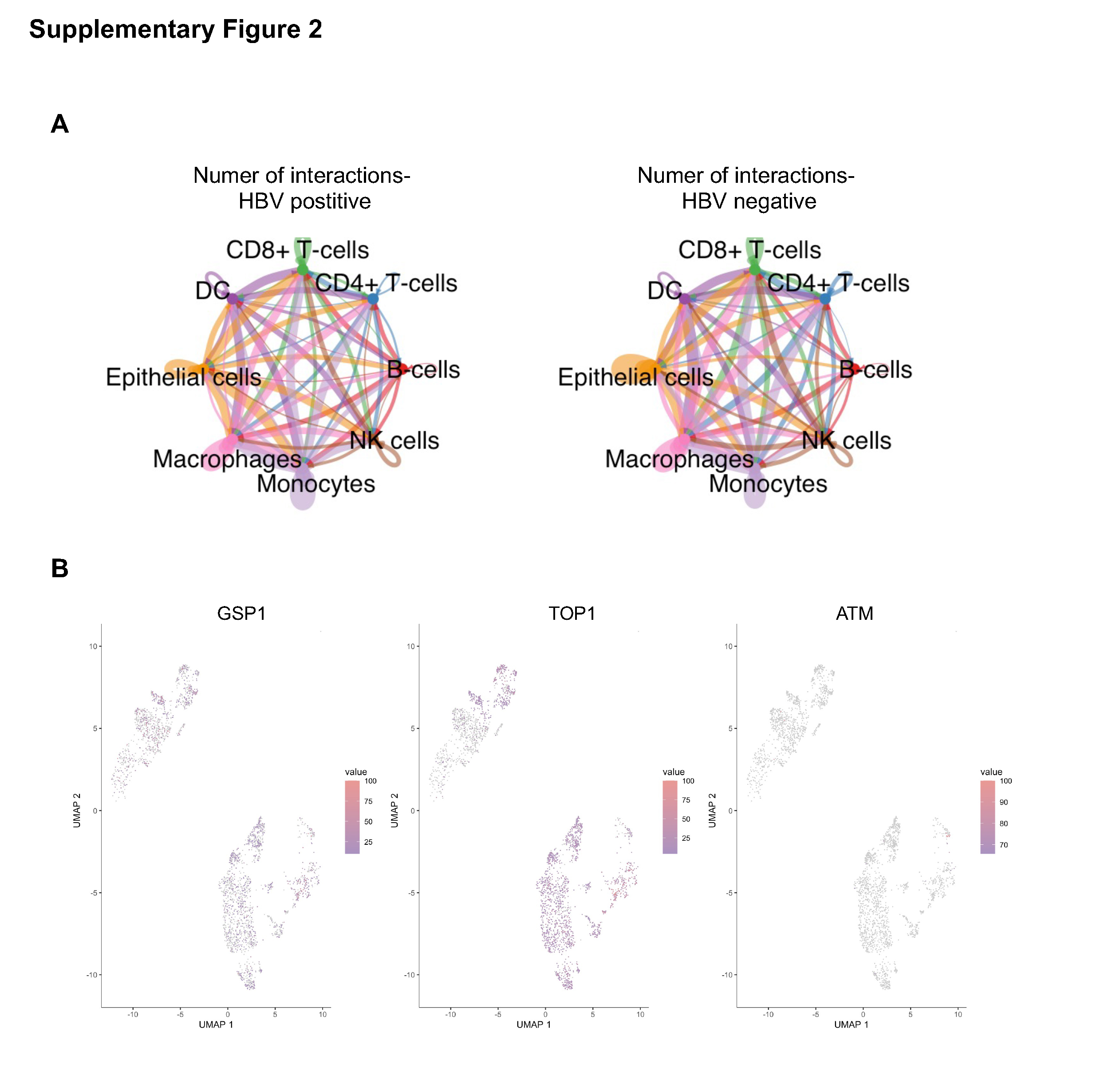

Supplement: Supplementary file 2 — Supporting Information [file CTM2-15-e70171-s009.jpg]

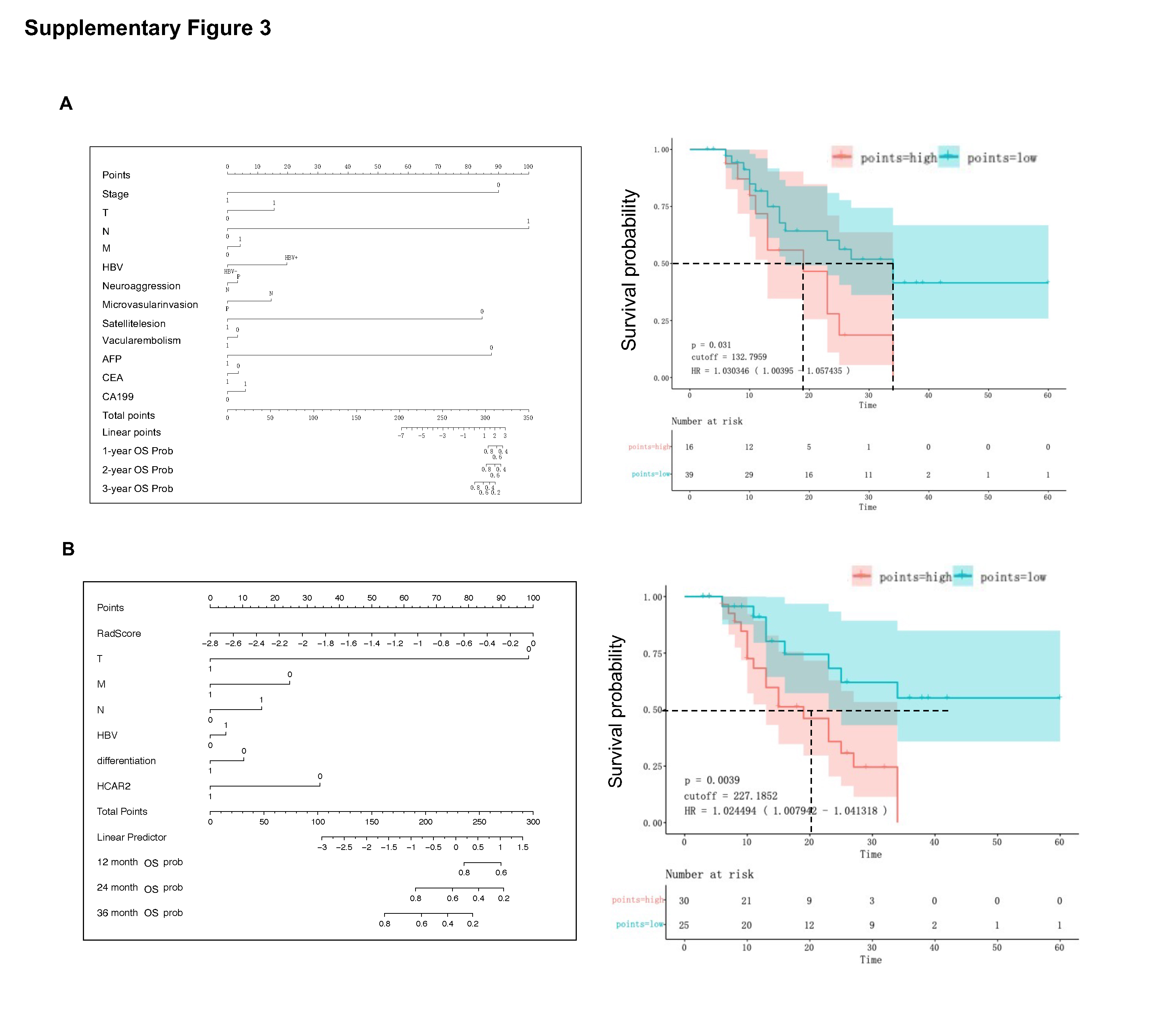

Supplement: Supplementary file 3 — Supporting Information [file CTM2-15-e70171-s005.jpg]
