# Supplementary material for: Predictive nomogram integrating radiomics and multi‐omics for improved prognosis‐model in cholangiocarcinoma
Source: Clin Transl Med. 2025 Jan 12;15(1):e70171. doi: 10.1002/ctm2.70171 (PMC11726632; doi:10.1002/ctm2.70171)
Supplement: Supplementary file 4 — Supporting Information [file CTM2-15-e70171-s010.docx]

| Characteristics | Training Cohort  (n = 210) | Internal Validation Cohort (n = 90) | External Validation Cohort (n = 89) | *P value* |
| --- | --- | --- | --- | --- |
| Age | 62.33±8.97 | 60.93±8.90 | 59.73±9.97 | 0.188 |
| Male, n/total (%) | 118/210(63.1) | 55/90(68.8) | 53/80 (66.2) | 0.456 |
| Total bilirubin(μmol/L) | 69.17[54.14-84.20] | 59.77[40.68-78.85] | 69.17[54.14-84.20] | 0.605 |
| Albumin(g/L) | 41.29[40.50-42.08] | 39.81[38.68-41.24] | 38.81[30.68-45.04] | 0.077 |
| ALT(U/L) | 91.66[71.83-111.49] | 100.92[73.2-128.64] | 89.81[68.68-111.24] | 0.265 |
| AST(U/L) | 72.98[57.26-88.70] | 81.82[58.60-105.14] | 77.82[51.00-95.34] | 0.602 |
| AFP ≤ 20, (%)(ng/ml) | 179(85.2) | 75(83.7) | 79(88.7) | 0.390 |
| CEA ≤5, (%) (ng/ml) | 131(62.1) | 58(64.5) | 56(63.1) | 0.271 |
| CA-199 ≤35, (%)( U/ml) | 116(55.0) | 52(57.0) | 50(56.0) | 0.163 |
| Ferritin ≤ 323, (%) | 84(44.9) | 34(42.5) | 36(41.5) | 0.282 |
| PIVKA-II High, (%) | 17(9.1) | 7(8.8) | 10(9.8) | 0.988 |
| Hepatits B virus, total (%) | 43(23.0) | 25(31.3) | 20(29.3) | 0.158 |
| Vascular invasion, total (%) | 15(8.0) | 7(8.8) | 8(8.9) | 0.585 |
| Perineural invasion | 70(37.4) | 22(27.5) | 28(31.5) | 0.294 |
| Vessel cancer embolus, total (%) | 37(19.8) | 6(7.4) | 8(10.4) | 0.044 |
| Satellite lesions, total (%) | 5(2.7) | 3(3.8) | 3(3.0) | 0.535 |
| pT-classification, total (%) | 120(64.2) | 57(71.3) | 61(72.3) | 0.532 |
| pN-classification, total (%) | 69(36.9) | 38(47.5) | 35 (42.5) | 0.238 |
| pM-classification, total (%) | 103(55.1) | 43(53.8) | 45(55.8) | 0.246 |
| AJCC 8th TNM, total (%) | 38(20.3) | 24(30.0) | 26(27.0) | 0.455 |

**Table 1. Clinical characteristics of ICC in the training and internal and validation cohorts**
