# Supplementary material for: Predictive nomogram integrating radiomics and multi‐omics for improved prognosis‐model in cholangiocarcinoma
Source: Clin Transl Med. 2025 Jan 12;15(1):e70171. doi: 10.1002/ctm2.70171 (PMC11726632; doi:10.1002/ctm2.70171)
Supplement: Supplementary file 11 — Supporting Information [file CTM2-15-e70171-s003.docx]

**Supplementary methods**

## **Participants**

This study received approval from the Institutional Review Board (the First Affiliated Hospital, Zhejiang University School of Medicine.) and waived the requirement for written informed consent for this retrospective review. The study was conducted in accordance with the Helsinki Declaration. From January 2014 to March 2021, a total of 389 patients with histopathologically confirmed ICC, were retrospectively enrolled across three institutions: the First Affiliated Hospital, Zhejiang University School of Medicine (Institute 1), the Second Affiliated Hospital, Zhejiang University School of Medicine (Institute 2), and the Affiliated Southern Medical University Hospital (Institute 3). These patients were then assigned to either the training cohort (n = 210), the internal validation cohort (n = 90) or external validation cohort (n = 89), drawn from different institutions or time periods, following a 7:3 allocation ratio. Among them, 20 subjects were excluded for the following reasons: 1) Pathologically combined with hepatocellular carcinoma (HCC) and ICC (n = 36); 2) Unsatisfactory image quality (n = 7).

## **Clinical Data and Pathological Assessment**

Clinical information was collected from the electronic medical record system. In addition to general information (gender and age), gallbladder stones, liver function indicators (albumin (ALB), gamma-glutamyl transferase (GGT), total bilirubin (TBIL), alanine aminotransferase (ALT)), and tumor biomarkers (microRNA-7, Chitinase-3-like Protein 1 (CHI3L1), Protein Induced by Vitamin K Absence/Inhibition-II, ferritin level, alpha-fetoprotein (AFP), cancer antigen 19-9 (CA19-9), cancer antigen 125 (CA125) and carcinoembryonic antigen (CEA)) were retrospectively collected. Before surgery, all patients were tested for HBV-related factors, including hepatitis B surface antigen (HBsAg), hepatitis B e antigen (HBeAg), hepatitis B e antibody (HBeAb), and hepatitis B core antibody (HBcAb). The presence of MVI, cholecystolithiasis, perineural invasion, intravascular tumor thrombi, satellite nodules, tumor stage and tumor differentiation, was obtained from postoperative pathological reports.

## **Tumor Segmentation**

The MRI image were scrutinized by two radiologists with 5 years (doctor 1, D.W.) and 15 years (doctor 2, L.X.) of diagnostic imaging experience. All T1-weighted MRI images in DICOM format, original size and resolution were transferred to ITK-SNAP software (Version 3.8, www.itksnap.org) for three-dimensional (3D) region of interest (ROI) segmentation. Professional radiologists utilized ITK-SNAP software to perform 3D ROI segmentation on T1-weighted MRI images to precisely define tumor boundaries. Through repeated feature extraction by two radiologists with varying levels of experience on a subset of samples, the reliability and consistency of the features were assessed using the Intraclass Correlation Coefficient (ICC), ensuring the credibility of the study results. Additionally, to enhance analytical accuracy, data potentially affected by the partial volume effect was excluded.

**Imaging Technology**

The study employed a sophisticated 3.0 Tesla Magnetic Resonance Imaging (MRI) system, specifically a Discovery MR750 manufactured by GE Healthcare located in Milwaukee, USA. All MRI scans were executed with an eight-channel phased-array torso coil provided by GE Healthcare Systems to enhance image quality and resolution. Conventional MRI scanning included respiratory-gated Fast Spin-Echo (FSE) sequences. For fat-suppressed axial T2-weighted images, the Repetition Time (TR) and Echo Time (TE) were set to 3529 milliseconds and 77.6 milliseconds (effective), respectively. The slices were 5.0 millimeters thick with a 1 millimeter gap between them. The imaging field of view covered an area of 38×38 square centimeters, while the matrix size was configured at 320×320 pixels. Two signal averages (NEX 2.0) were used to improve signal-to-noise ratio. Contrast-enhanced T1-weighted axial images were acquired with a shorter TR/TE of 3.9/1.8 milliseconds (effective), maintaining the same slice thickness and gap as T2-weighted images. The field of view was adjusted to 38×30.4 square centimeters, with a matrix size of 320×160 pixels, and a single signal average (NEX 1) was applied. The entire sequence of conventional MRI scans took roughly 15 minutes to complete. Intravoxel Incoherent Motion (IVIM) analysis was conducted utilizing Echo-Planar Imaging (EPI) in the axial plane, also incorporating respiratory gating. Parallel imaging techniques were employed with parameters configured as: TR/TE of 3750/61.4 milliseconds; field of view of 38×28 square centimeters; a matrix size of 128×128; and a slice thickness of 5.0 millimeters with a 1-millimeter gap. A range of 13 b-values were used to measure diffusion characteristics, starting from 0 up to 1200 seconds per square millimeter squared (specifically 0, 10, 20, 40, 80, 100, 150, 200, 400, 600, 800, 1000, 1200 seconds/mm²), with varying numbers of signal averages (NEX) depending on the b-value intensity, ranging from 2 to 10 repeats for the highest b-value. The entire IVIM acquisition process took around 10 minutes. This comprehensive MRI protocol aimed to provide detailed anatomical and functional information about the scanned tissues, particularly focusing on diffusion properties, which can be critical in diagnosing and monitoring various diseases.

## **Radiomics Feature Extraction**

Prior to radiomics feature extraction, to reduce feature variability, we performed the following image preprocessing steps, containing gray discretization, intensity normalization and voxel resampling. Then, radiomics features were extracted from T1WI MRI images through the open source PyRadiomics library. They are divided into four categories: size and morphology features, descriptors of image intensity histograms, descriptors of the relationship between image voxels and higher-order texture features extracted from filtered images. In order to prevent signature overfitting, dimensionality of features is reduced before signature construction. Succinctly, radiomics features that met the inter-observer and intra-observer ICCs criteria greater than 0.8 and were significantly different between the two groups as assessed by ANOVA were included in the LASSO regression model to select the most valuable features in the training set. Finally, the selected radiomics features were used to construct radiomics signature. Rad-score was calculated for each patient by linear combination of selected features and weighted by the respective LASSO coefficients.

## **Construction of Machine Learning Radiomic-model**

For the features obtained from the training and validation sets, we applied Lasso regression for imaging feature extraction and aimed to identify the most predictive key features from a given feature set. Subsequently, utilizing the glmnet package in the R programming language, we perform 100-fold cross-validation to extract imaging features pertinent to clinical indicators, mutated genes, or expressed proteins of interest. This process is fundamental for the construction of subsequent machine learning models. Based on the model developed using the Lasso regression methodology, we calculate imaging scores (RadScore) for two groups differentiated by gene mutations or gene expression of interest in exon or proteome data. To assess the model's discriminatory power between the two sample groups, we employ the Wilcoxon rank-sum test and present the results through box plots and bar charts for visual analysis. Furthermore, to evaluate the overall performance of our classifier, we utilize the pROC package in R to calculate the Area Under the Curve (AUC) and construct the corresponding ROC curve.

## **Multi-Omics Data Analysis**

In the multi-omics analysis of ICC, we comprehensively utilized whole-exome sequencing (WES), proteomics, and single-cell RNA sequencing (scRNA-seq) techniques. In the WES analysis, we employed advanced variant detection technology to identify SNPs and INDELs, and conducted exhaustive functional annotation and prioritization of variants through comprehensive comparison with public databases and disease-related databases. The selection of potential driver mutations is primarily based on the following criteria: We prioritize high-frequency or recurrent mutations that appear across multiple samples. We reference published research, especially studies involving similar cancer types, to identify mutations that have been proven to be associated with cancer development. Simultaneously, we perform pathway enrichment analysis using databases such as KEGG and Reactome to identify mutations enriched in key signaling pathways. Additionally, we integrate clinical data from patients, including tumor staging, treatment responses, and prognostic information, to select mutations significantly correlated with clinical phenotypes. At the proteomics level, we used mass spectrometry techniques to deeply analyze the protein expression profiles in ICC patient samples. Through precise peak identification and quantitative analysis, we identified the differential expression of key proteins and further revealed their potential roles in disease progression through pathway analysis.

**scRNA-seq Analysis of HBV-negative and HBV-positive ICC**

***scRNA-seq Library Preparation and Sequencing***

Single-cell suspensions were counted using a Countess™ automated cell counter (Thermo Fisher Scientific) and adjusted to 1000 cells/mL. Cells were loaded according to the Chromium Single Cell Kit standard protocol, targeting 5,000-10,000 cells per chip position (V2 chemistry). Library construction and all subsequent procedures were performed according to the manufacturer's standard protocols.

***scRNA-seq Data Sequencing and Processing***

Single-cell libraries were sequenced on an Illumina HiSeq X Ten using 150 nt paired-end sequencing. Cell Ranger (version 2.2.0) was used to process raw data, demultiplex cell barcodes, map reads to the transcriptome, and downsample reads (as needed to generate normalized aggregate data across samples). These processes produced a raw unique molecular identifier (UMI) count matrix, which was converted into a Seurat object using the R package Seurat (version 5.1.0). Cells with < 1000 UMIs were considered low-quality and removed. To eliminate potential doublets, single cells with > 4000 detected genes were also filtered out. After removing low-quality cells and doublets, a total of 7,748 cells were retained for downstream analysis. The filtered gene-cell matrix was normalized for library size in Seurat to obtain normalized UMI count data.

***scRNA-seq Data Cell Clustering***

Normalization and variable gene selection were performed using SCTransform (https://github.com/ChristophH/sctransform). Seurat was applied to the normalized gene-cell matrix to identify highly variable genes for unsupervised cell clustering. To identify highly variable genes, the MeanVarPlot method in the Seurat package was used to establish the mean-variance relationship of normalized counts for each gene across cells. To reduce the dimensionality of the scRNA-Seq dataset, principal component analysis (PCA) was performed on the integrated data matrix, with the first 20 PCs used for downstream analysis. Major cell clusters were identified using Seurat's FindClusters function with a resolution of 0.1. Clusters were visualized using 2D tSNE or UMAP plots.

***Identification of Major Cell Types in scRNA-seq Data***

To define the major cell type of each single cell, differentially expressed genes (DEGs) for each cell cluster in ICC1 were identified using the FindAllMarkers analysis in the Seurat package, and the top 20 most significant DEGs were carefully analyzed. Simultaneously, feature plots for the top 20 marker genes of each subgroup were generated, GO term enrichment analysis was performed, and two representative GO terms for each subgroup were extracted.

***Trajectory Inference of scRNA-seq Data Cells***

To map the differentiation spectrum in the tumor microenvironment (TME), we performed pseudotime analysis using Monocle3 to determine significant transitional relationships between cell types and cell clusters. Specifically:Pre-computed UMAP results were extracted from the Seurat object for the ICC1 sample and filtered for specific cell types, including B cells, CD4+ T cells, CD8+ T cells, dendritic cells (DCs), epithelial cells, hematopoietic stem cells (HSCs), macrophages, monocytes, and natural killer (NK) cells; Cells were clustered using Monocle3's cluster_cells function and annotated using either Seurat clustering results or cell type labels.The cells were then ordered in pseudotime using Monocle3's order_cells function, and cell trajectory plots were visualized using the plot_cells function; Genes that change significantly over pseudotime were identified using the top_markers function, and trends of temporally related genes were displayed using the plot_genes_in_pseudotime function; Developmental relationships in the context of annotated cell types and pseudotime gradients were presented using UMAP plots, and cell trajectory plots were generated using the plot_cells function. Ligand-receptor analysis and visualization were performed using CellChat v2.1.2 (github.com/sqjin/CellChat). Cell type labels from the cell_type_bpe column of the Seurat object were used. Default parameters were used for each step.

## **Prediction Model Construction and Survival Analysis Method**

The following three models were built in this study: clinicoradiological, radiomics, and clinical-genomic-radiomics models. To construct the clinicoradiological model, univariate regression was first used to analyze radiological and clinical characteristics, such as sex, age, and intratumoral necrosis. Significant variables were further selected for the multivariate regression model. All Based on clinically relevant indicators/RadScore (a radiological score constructed for HBV models) and gene mutation status, a Cox proportional hazards regression model is constructed using R software. The nomogram function within R is then utilized to create a nomogram (Nomogram chart). Utilizing the overall scores obtained for each individual based on this method, R software's surv_cutpoint package is used to determine the optimal cutoff value. With this cutoff, alongside the subjects' survival time and outcomes, survival analysis is performed. Subsequently, the ggsurvplot package in R is employed to visualize the results of the survival analysis through survival curves. In the predictive model, the scale along each line segment corresponding to a variable represents the range of possible values for that variable. The individual point score for each variable corresponds to its respective item score; summing up all the variables' scores gives the Total Points. The resulting total score directly corresponds to the predicted probability of OS at 12, 24, and 36 months. Using the optimal cutoff value obtained, the same method is applied to draw survival curves in a validation set of 88 samples.

## **Statistical Analysis**

Subsequent analysis was performed using R version 3.2.3 (R Foundation for Statistical Computing). All radiomic features (n = 4,842) were normalized by transforming the data into new scores with a mean of 0 and a SD of 1 (z-score transformation). Patients were randomly allocated to a training and validation set (2:1 ratio with n = 210 patients in the training and n = 89 patients in the validation set) with the distribution of survival rates kept balanced between both sets.

# **﻿Supplementary Figure Legends**

**Supplementary Figure 1.**

**A, B**. Receiver Operating Characteristic (ROC) curves for the Random Forest (RF), Logistic Regression (LR), and Support Vector Machines (SVM) models constructed to predict various pathological factors and gene mutations. The ROC curves are shown separately for the training cohort, internal validation cohort, and external validation cohort. Specifically, the factors evaluated include CA-125, CA-199 levels, neuroinvasion (A), and mutations in KRAS, BRAF, and FGFR2 genes (B). The performance of the models is evaluated by calculating the Area Under the Curve (AUC), sensitivity, specificity, accuracy, and precision. **C.** The most frequently detected driver gene variants associated with HBV status, including USP17L7, MUC4, USP17L2, ZNF99, HNRNPL2, NBPF12, HCAR2, and TRIM49. **D**. Mutation rate of the MUC4 gene in the TCGA database, with approximately 21% of analyzed tumor samples carrying mutations. **E**. Higher tumor mutation burdens (TMB) and mutation counts in ICC patients harboring mutations in specific genes, including USP17L7, ZNF99, HCAR2, and TRIM49. **F.** Proteomic-based gene set enrichment analysis revealing significantly upregulated oncogenic pathways in HBV-positive ICCs. **G**. Receiver Operating Characteristic (ROC) curves demonstrating the ability of the radiomic model to distinguish HBV+ from HBV- status in the training cohort (AUC = 0.73), internal validation cohort (AUC = 0.64), and external validation cohort (AUC = 0.65). **H**. ROC curves demonstrating the correlation between radiomic features extracted from medical images and genomic markers (MUC1 / ESRP1 protein expression and MUC4 / USP17L7 gene mutation) in patients with HBV-infected ICC.

**Supplementary Figure 2**.

**A**. Differential analysis of intercellular interactions between HBV-ICC and non-HBV-ICC samples. **B**. Interactive visualization of the relative expression levels of GSTP1, TOP1, and ATM genes in the same UMAP coordinates.

**Supplementary Figure 3**.

**A**. The pathology-based OS model presented with a nomogram scaled by the proportional regression coefficient of each predictor. Kaplan-Meier survival curves for the pathology-based OS model. Patients are stratified into high-risk and low-risk groups based on the model cutoff value. The 1-year, 2-year, and 3-year survival rates are significantly different between the two groups. **B**. Kaplan-Meier survival curves for the imaging-pathology-genomic based OS model. Patients are stratified into high-risk and low-risk groups based on the model cutoff value. The 1-year, 2-year, and 3-year survival rates are significantly different between the two groups.
